# Mitochondrial base editor DdCBE causes substantial DNA off-target editing in nuclear genome of embryos

**DOI:** 10.1038/s41421-022-00391-5

**Published:** 2022-03-18

**Authors:** Yinghui Wei, Zhifang Li, Kui Xu, Hu Feng, Long Xie, Di Li, Zhenrui Zuo, Meiling Zhang, Chunlong Xu, Hui Yang, Erwei Zuo

**Affiliations:** 1 Shenzhen Branch, Guangdong Laboratory for Lingnan Modern Agriculture, Genome Analysis Laboratory of the Ministry of Agriculture, Agricultural Genomics Institute at Shenzhen, Chinese Academy of Agricultural Sciences, Shenzhen, Guangdong, China; 2 Institute of Neuroscience, State Key Laboratory of Neuroscience, Key Laboratory of Primate Neurobiology, CAS Center for Excellence in Brain Science and Intelligence Technology, Shanghai Institutes for Biological Sciences, Chinese Academy of Sciences, Shanghai, China; 3 International Peace Maternity and Child Health Hospital, School of Medicine, Shanghai Jiao Tong University, Shanghai, China; 4 Shanghai Center for Brain Science and Brain-Inspired Intelligence Technology, Lingang Laboratory, Shanghai, China

**Keywords:** Molecular biology, Bioinformatics

Dear Editor,

Mitochondrial DNA (mtDNA) is encapsulated by the organelle membrane^1^ forming the barrier for the access of CRISPR-based gene-editing tools to the mtDNA. Furthermore, mitochondrial genome lacks similar repair systems for the protection of nuclear genome from DNA damage after induction of double-strand break by programmable nuclease such as ZFN, TALEN etc., which results in the elimination of target mtDNA^2–5^ instead of mutation installation on mtDNA in contrast to the outcome of indel formation on nuclear DNA^6^. Recent studies positioned DdCBE as a promising technology to install targeted mutations or introduce transmissible mutations of base conversion in mammalian mtDNA^7–10^ rather than eliminate them with previous ZF- or TALE-based nuclease^2–5^. Thus, DdCBE has the potential to model mitochondrial disease mutations, correct pathogenic variants, and expand our knowledge of mitochondrial biology. However, it is worth mentioning that these studies have found that DdCBE can cause low-frequent off-target events on mtDNA^9,10^. As indicated, the off-target profile of DdCBE remained to be comprehensively investigated by additional research for their systematic effect on mtDNA as well as nuclear genome.

In the current study, we performed the GOTI (genome-wide off-target analysis by two-cell embryo injection) method previously developed by us^11,12^ to evaluate the off-target effect of DdCBE on both mtDNA and nuclear DNA modification. At first, we in vitro transcribed two pairs of DdCBE mRNA targeting the mtDNA *ND5* gene (G12918 and C12336) and injected them with Cre mRNA into one blastomere of two-cell embryos derived from Ai9 background leaving another blastomere uninjected (Fig. 1a and Supplementary Fig. S1a). Thereby, Cre-activated tdTomato fluorescence will distinguish DdCBE-injected cells from non-fluorescent uninjected cells derived from the same two-cell Ai9 embryos. At 14.5 days after transferring injected 2-cell embryos into surrogate female mice, we collected E14.5 embryos to sort tdTomato^+^ and tdTomato^–^ cells for genotyping base conversion outcomes on two targeted loci of mtDNA as well as whole-genome sequencing (WGS) analysis (Fig. 1a). Sanger sequencing and Targeted deep sequencing results showed efficient mtDNA editing by DdCBE with m.G12918A and m.C12336T conversion rate of up to 46% in both non-sorted and sorted tdTomato^+^ tissues, contrasting with the only wild-type alleles detected in tdTomato^–^ tissues (Supplementary Fig. S1b, c and Table S1). For G12918 and C12336-targeting DdCBE, we also observed higher editing rate of up to 72% for sorted cells than unsorted ones on the basis of WGS (Fig. 1b, c). Furthermore, there are several unintended and sequence-independent C-to-T editing events identified by WGS analysis with lower than 5% frequency centered around m.G12918A or m.C12336T on mtDNA (Supplementary Fig. S2a). For all unintended editing events, some fall within spacer sequence between two recognition sequences for the TALE pair while several reside out of the spacer sequence, indicating low-frequent and sequence-independent off-target editing on mtDNA for DdCBE (Fig. 1d, e). We further performed sequence enrichment analysis on off-target sites and revealed a cognate 5′-TC-3′ motif preference of DdCBE for non-specific sequences editing (Supplementary Fig. S2b). These results collectively demonstrate that DdCBE pairs can produce efficient on-target base editing and low-frequent non-specific editing near the target loci of mtDNA.

**Fig. 1:**
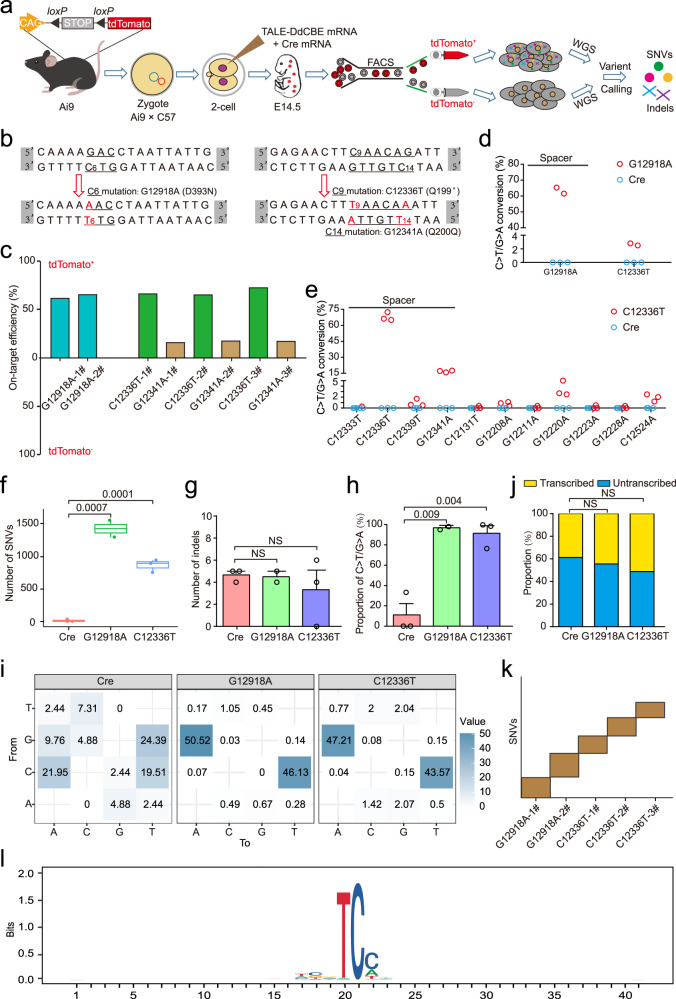
Off-target analysis of DdCBE for mitochondrial and nuclear genome editing with GOTI. **a** GOTI workflow for analyzing off-target profile of DdCBE. **b** The DdCBE target for generating the m.G12918A point mutation (D393N), m.C12336T nonsense mutation (Q199stop), and m.G12341A silent mutation (Q200Q) in the ND5 protein. Translation triplets are underlined and target sequences with possible editing loci are shown in red. **c** On-target efficiency of *ND5*-DdCBE (m.G12918A and m.C12336T) for tdTomato^+^ and tdTomato^–^ cells on the basis of WGS. Distribution pattern of off-target sites in m.G12918A (**d**) and m.C12336T (**e**) E14.5 fetuses (red dots) with Cre fetuses as control (blue dots). Spacer represents region between recognition sequences of the TALE pair. Each dotted box indicates a single off-target event. **f** Comparison of the total number of identified off-target SNVs in Cre and *ND5*-DdCBE (m.G12918A and m.C12336T) injected groups by WGS. **g** Number of indels identified in Cre and *ND5*-DdCBE (m.G12918A and m.C12336T) injected groups by WGS. **h** Proportion of C·G to T·A mutations among all identified SNVs for Cre and *ND5*-DdCBE (m.G12918A and m.C12336T) injected groups. **i** Distribution of mutation types. The number in each cell indicates the proportion of a certain type of mutation among all mutations. **j** The distribution of off-target SNVs in the transcribed and untranscribed regions. **k** SNVs identified from all DdCBE-edited samples did not overlap, suggesting that off-targets on the nuclear genome were mainly caused by the sequence-independent activity of DdCBE. **l** Sequence logos generated from off-target sequences with C·G to T·A conversions by *ND5*-DdCBE in nuclear genome. Bits reflect sequence conservation at a given position. Data are presented as the means ± SEM. *P* values were evaluated with the unpaired Student’s *t*-test (two-tailed).

To evaluate the potential influence of DdCBE activity on nuclear DNA in mammalian cells, we performed WGS for both edited cells marked by tdTomato fluorescence and unedited cells derived from the same embryo created as the previous GOTI protocol^11,12^. Unexpectedly, we identified about 1500 and 1000 single-nucleotide variants (SNVs) with significant confidence as potential off-target editing events for G12918 and C12336-targeting DdCBE in edited cells using unedited cells as SNV calling references (Fig. 1f). To exclude the possibility of SNVs caused by Cre injection, we also analyzed the SNV number for Cre-only samples without DdCBE injection and found less than 20 SNVs in Cre-only samples (Fig. 1f). In addition to SNVs analysis, indel (insertion and deletion) frequency was further checked in WGS datasets with less than 5 indels detected for both Cre-only and Cre-plus-DdCBE samples (Fig. 1g). Our results showed that DdCBE could significantly increase SNVs formation in edited cells while exhibit undetectable effect on indel frequency (Fig. 1f, g). Since SNVs identified by GOTI showed random distribution in nuclear genome^11,12^, we analyzed all SNVs identified in G12918 and C12336-targeting samples to find significantly strong enrichment of C-to-T/G-to-A conversion among five different base conversion outcomes, consistent with cytosine deaminase activity of DdCBE (Fig. 1h, i and Supplementary Fig. S3a). In contrast, SNVs identified in Cre-only samples exhibited no enrichment for any type of base conversion, corroborating off-target activity of DdCBE on nuclear DNA (Fig. 1h, i and Supplementary Fig. S3a). By examining SNVs distribution within transcribed and untranscribed regions, we found non-significant correlation of DdCBE-affected SNVs with genomic transcription features similar to the trend found in Cre-only samples (Fig. 1j). Moreover, off-target SNVs caused by DdCBE exhibited even and random distribution in the entire genome as SNVs identified in Cre-only samples (Supplementary Fig. S3b). In addition, there are no overlapping SNVs among all DdCBE-edited samples, suggesting sequence-independent off-target activity of DdCBE (Fig. 1k). Besides, we also checked the potential sequence-dependent off-target bias towards nuclear mitochondrial DNA segments (NUMTs) or other sequence similar to *ND5* target loci. Our analysis revealed neither off-target editing nor efficiency bias towards similar sequences or NUMTs contrasted to the majority of off-target sequences (Supplementary Fig. S4). Lastly, we put together off-target sequences identified in all DdCBE-edited samples to find again the enrichment of 5′-TC-3′ motif for DdCBE-affected SNVs as the results on mitochondrial DNA (Fig. 1l). To verify SNVs identified in DdCBE-edited samples, we performed Sanger sequencing on random selected regions and found all of regions were edited in tdTomato^+^ cells while remained unedited in tdTomato^–^ cells (Supplementary Fig. S5). Overall, our GOTI results demonstrated notable sequence-independent off-target activity of DdCBE on nuclear DNA of edited tissue derived from DdCBE-injected blastomere.

In summary, we showed for the first time that DdCBE cause thousands of off-target SNVs enriched for C-to-T/G-to-A conversion in the entire nuclear genome, which is two times the SNV number resulted from low-fidelity base editor BE3. Unlike the substrate preference of cytosine deaminase APOBEC1 in BE3 protein for ssDNA, DddA_tox_ in DdCBE protein is a unique type of cytosine deaminase with dsDNA as substrate^7^. It might explain more off-target SNVs observed for DdCBE than BE3. Since DdCBE was designed to localize in mitochondria guided by mitochondrial targeting signal (MTS) in N-terminal region of DdCBE protein, our results imply that MTS seems to fail in blocking the entry of DdCBE into nuclear in mammalian cells. It would be interesting in the future to examine whether extra or different MTS could reduce off-target editing of DdCBE on nuclear DNA. In addition, expressing DdCBE in embryos may result in different results from when editing is done in differentiated cells. Therefore, it would be necessary when the technique available in the future to clarify different propensity of DdCBE off-target activity in somatic cells than embryonic cells. Taken together, our finding on off-target activity of DdCBE towards nuclear genome necessitate the strong need to optimize DdCBE for specific base editing on mtDNA especially before being used for treating mitochondrial diseases.

## Supplementary information

Supplementary Figures and Tables

## Data Availability

The whole-genome sequencing data have been deposited to the NCBI Sequence Read Archive (SRA) database (accession ID, PRJNA786071).
