# Supplementary material for: Mitochondrial base editor DdCBE causes substantial DNA off-target editing in nuclear genome of embryos
Source: Cell Discov. 2022 Mar 18;8:27. doi: 10.1038/s41421-022-00391-5 (PMC8933521; doi:10.1038/s41421-022-00391-5)
Supplement: Supplementary file 1 — Supplementary Figures and Tables [file 41421_2022_391_MOESM1_ESM.pdf]

Supplementary information

Mitochondrial base editor DdCBE cause substantial DNA off-target editing in nuclear genome of embryos

Yinghui Wei[1,2], Zhifang Li[1], Kui Xu[1], Hu Feng[1], Long Xie[1], Di Li[1], Zhenrui Zuo[1], Meiling Zhang[4], Chunlong Xu[3], Hui Yang[2,3], Erwei Zuo[1]

Contents

Materials and Methods

Supplementary figures and legends

Supplementary Table S1

Supplementary Table S2

Supplementary sequence

Materials and Methods

Experimental design

The mixture of Cre and DdCBE mRNA was injected into one blastomere of a 2-cell embryo derived from mating Ai9 male mice with wild-type C57BL/6 female mice. The action of Cre is expected to generate a chimeric embryo with half of cells labeled with tdTomato (colored red). The tdTomato$^+$ cells and tdTomato$^-$ cells were isolated from the chimeric embryo at E14.5 by FACS for WGS. The off-target SNVs and indels were identified by comparing the tdTomato$^+$ with tdTomato$^-$ cells using three variant calling algorithms as indicated (Mutect2, Lofreq and Strelka for SNVs, and Mutect2, Scalpel and Strelka for indels). SNVs and indels were depicted as colored dots and crosses respectively in Fig. 1a.

Animals

Experiments involving mice were approved by the Biomedical Research Ethics Committee of Center for Excellence in Brain Science and Intelligence Technology/Institutes of Neuroscience, Chinese Academy of Sciences. Super ovulated C57BL/6 females (4 weeks old) were mated with heterozygous Ai9 males (full name B6.Cg-Gt (ROSA) 26Sortm9 (CAG-td-Tomato) Hze/J; JAX strain 007909), and females from the ICR strain were used as foster mothers. Mice were maintained in a specific pathogen-free facility under a 12-hour dark–light cycle, and constant temperature (20–26°C) and humidity (40–60%) maintenance.

In *vitro* transcription of DdCBE and Cre mRNA

The *ND5*-DdCBE (m.G12918A and m.C12336T) plasmids were obtained from Addgene deposited by Dr. Jin-Soo Kim lab[1] and protein sequences encoding DdCBE used in the study were provided in Supplementary sequence. The DdCBE plasmids were linearized and used as the template for *in vitro* transcription using the mMESSAGE mMACHINE T7 Ultra kit (Life Technologies). The DdCBE mRNA were purified using the MEGAclear kit (Life Technologies) and eluted in RNase-free water. *In vitro* transcribed RNAs were aliquoted and stored at -80°C until use. Prior to

microinjection, the DdCBE mRNA was prepared by centrifuge for 10 min at 14,000 rpm at 4°C and supernatant transferred to 0.2 mL fresh PCR tubes for injection. Cre mRNA was similarly prepared with the DdCBE mRNA as described here.

Microinjection of mouse 2-cell embryos with DdCBE mRNA

Super ovulated C57BL/6 females (4 weeks old) were mated with heterozygous Ai9 males (full name B6.Cg-Gt(ROSA)26Sortm9(CAG-td-Tomato)Hze/J; JAX strain 007909), and fertilized embryos were collected from oviducts 23 h post hCG injection. For 2-cell injection, the mixture of left TALE-DdCBE (100 ng/μL) and right TALE-DdCBE mRNA (100 ng/μL) was injected with Cre mRNA (5 ng/μL) into the cytoplasm of one blastomere of 2-cell embryo 48 h post hCG injection in a droplet of M2 medium containing 5 μg/mL cytochalasin B (CB) using a FemtoJet microinjector (Eppendorf) with constant flow settings. The injected embryos were cultured in KSOM medium with amino acids at 37°C under 5% CO_2 in air for 2 hours and then transferred into oviducts of pseudo-pregnant ICR foster mothers at 0.5-d.p.c.

Fluorescence activated Cell Sorting (FACS)

To isolate tdTomato$^+$ with tdTomato$^-$ cells, the whole E14.5 embryos were dissected and dissociated enzymatically in 5 mL Trypsin-EDTA (0.05%) at 37°C for 30 min. The enzymatic dissociation was stopped by adding 5 mL of DMEM medium with 10% Fetal Bovine Serum (FBS). Fetal tissues were then homogenized by passing 30–40 times through a 1 mL pipette tips. The cell suspension was centrifuged for 6 min at 800 rpm, and the pellet was resuspended in DMEM medium with 10% FBS. Finally, the cell suspension was filtered through a 40-μm cell strainer, and tdtomato$^+$/tdtomato$^-$ cells were isolated by FACS. Samples for WGS analysis were found to be >95% pure when assessed with a second round of flow cytometry and fluorescence microscopy analysis.

Whole genome sequencing (WGS) and data analysis

WGS protocol for GOTI was described in the previous study[2]. Briefly, genomic DNA was extracted from sorted tdTomato$^+$ or tdTomato$^-$ cells using the DNeasy blood and

tissue kit (Qiagen) according to the manufacturer's instructions. WGS was performed at the mean coverage of 50× by Illumina NovaSeq 6000 platform. Trimmomatic (v0.39) was used to trim the low quality reads and adapter sequences in the FASTQ files. Qualified reads were mapped to the mouse reference genome (mm10) by BWA (v0.7.12) with mem -M and Picard-tools (v2.3.0) was used to reorder, sort, add read groups and mark duplicates of the aligned BAM files. Then, Strelka (v2.7.1), Lofreq (v2.1.2) and Mutect2 (v4.1.5) were used to identify the genome wide de novo variants with high confidence. In parallel, Mutect2 (v4.1.5), Scalpel (v0.5.3) and Strelka (v2.7.1) were run individually for the detection of whole genome *de novo* indels. Only SNVs or indels identified by all the three algorithms were used for the following analysis. The variants were identified in the mapped BAM file of tdTomato$^+$ sample, with the tdTomato$^-$ sample in the same embryo as control and only variants mutated in the tdTomato$^+$ sample could be identified. For example, if the WT allele is G in a certain coordinate, the tdTomato$^+$ cells carries A, and the tdTomato$^-$ cells carries G, then the mutant A will be called as a *de novo* mutation. If tdTomato$^-$ cells carries A, the mutant could not be identified. To further validate the off-target SNVs were exclusively identified in tdTomato$^+$ samples, we also called variants in the tdTomato$^-$ sample with the tdTomato$^+$ sample in the same embryo as control, in which only variants mutated in the tdTomato$^-$ cells but were WT in the tdTomato$^+$ cells could be identified. To strictly control the quality of the variants, we removed variants overlapped with UCSC repeat regions and microsatellite sequences or reported in dbSNP151 database.

Genotyping analysis

The genotypes or off-target analysis of mutant E14.5 embryos were determined by PCR of genomic DNA extracted from tdTomato$^+$ and tdTomato$^-$ cells. Phanta Max Super-Fidelity DNA Polymerase (Vazyme Biotech Co., Ltd) was activated at 95°C for 5 min, and PCR was performed for 35 cycles at 95°C for 30 sec, 60°C for 30 sec, and 72°C for 30s, with a final extension at 72°C for 5 min. Subsequently, PCR products were subjected to Sanger or Targeted deep sequencing. PCR primers for genotype

analysis are indicated in Supplementary Table S2.

Targeted deep sequencing

Target sites were amplified by nested PCR from genomic DNA using Phanta Max Super-Fidelity DNA Polymerase (Vazyme Biotech Co., Ltd). The paired-end sequencing of PCR amplicons was performed by GENEWIZ Co., Ltd using NovaSeq 6000 platform. The sequencing data were subsequently demultiplexed using fastq-multx (v1.4.1) with the PCR primers. We next performed sequence alignment between the demultiplexed sequencing data with each of the on-target and off-target sites using CRISPResso2 (v2.0.32), and then generate mapping statistics using in-house scripts with Perl (v5.26.2) and R (v4.1.0).

Statistical analysis

All statistical values were presented as means ± SEM. Differences between datasets were considered to be significant at *P* value less than 0.05. All statistical tests were conducted with the unpaired student's *t*-test (two-tailed), unless otherwise stated.

References

1 Lee, H. et al. Mitochondrial DNA editing in mice with DddA-TALE fusion deaminases. *Nat. Commun.* **12**, 1190 (2021).

2 Zuo, E. et al. GOTI, a method to identify genome-wide off-target effects of genome editing in mouse embryos. *Nat. Protoc.* **15**, 3009-3029 (2020).

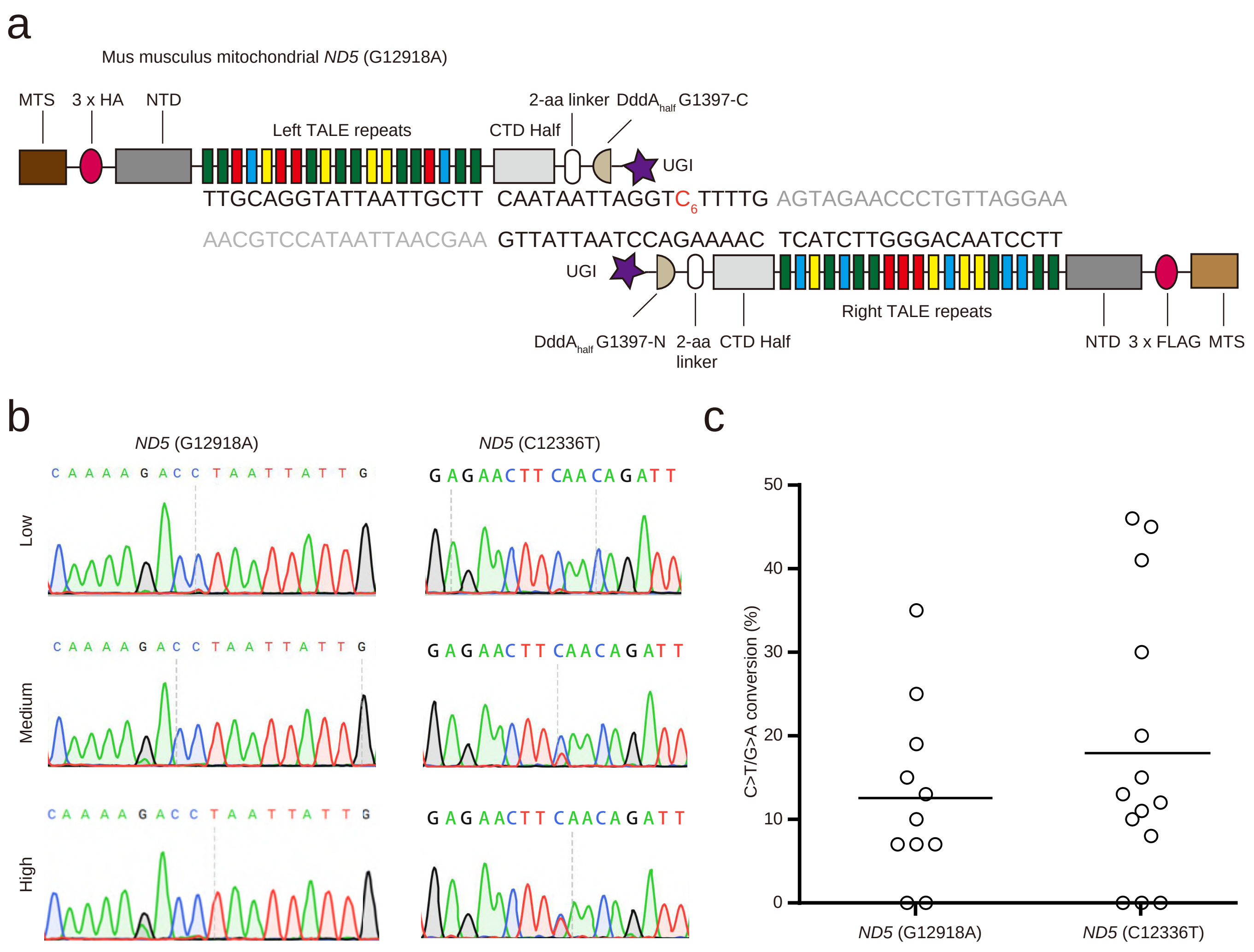
a
Mus musculus mitochondrial ND5 (G12918A)
MTS
3 x HA
NTD
2-aa linker
DddAhalf G1397-C
Left TALE repeats
CTD Half
UGI
TTGCAGGTATTAATTGCTT CAATAATTAGGTC6TTTTG AGTAGAACCCTGTTAGGAA
AACGTCCATAATTAACGAA GTTATTAATCCAGAAAAC TCATCTTGGGACAATCCTT
UGI
Right TALE repeats
DddAhalf G1397-N
2-aa linker
CTD Half
NTD
3 x FLAG
MTS
b
ND5 (G12918A)
ND5 (C12336T)
Low
Medium
High
C A A A A G A C C T A A T T A T T G
G A G A A C T T C A A C A G A T T
c
C>T/G>A conversion (%)
0
10
20
30
40
50
ND5 (G12918A)
ND5 (C12336T)

Supplementary Fig. S1. DdCBE mediated on-target editing efficiency in E14.5 embryos. a. Schematic of DdCBE targeting *ND5* (m.G12918A) gene in mitochondrial genome. **b.** Representative Sanger sequencing chromatograms of low (upper), medium (middle) and high (lower) mutation loads in m.G12918A and m.C12336T E14.5 fetuses without FACS. **c.** Mutation loads of edited mice obtained through microinjection of DdCBE targeting G12918 (n=11) and C12336 (n=14).

a

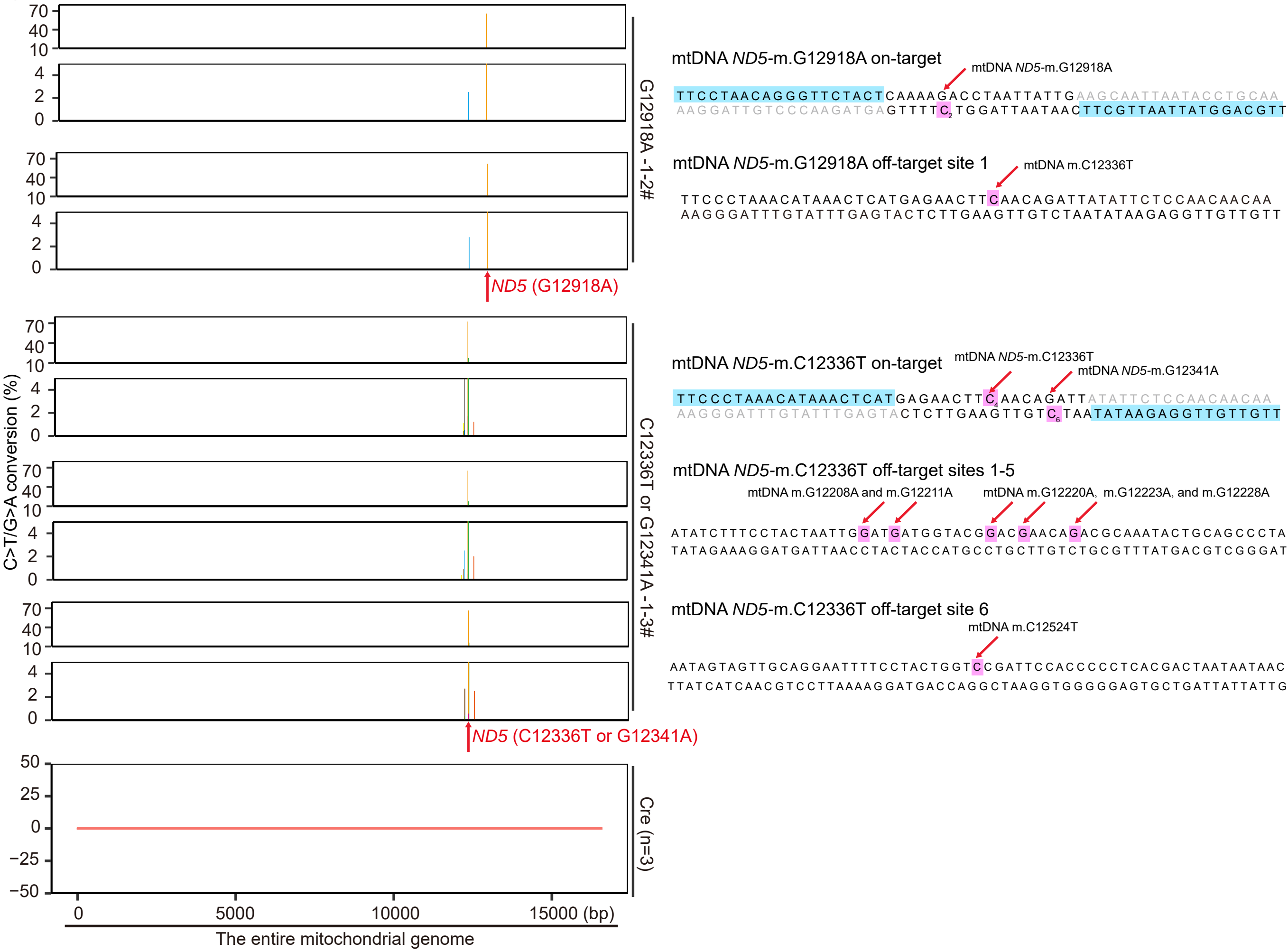

C>T/G>A conversion (%)
70
40
10
4
2
0
G12918A -1-2#
ND5 (G12918A)
C12336T or G12341A -1-3#
ND5 (C12336T or G12341A)
50
25
0
−25
−50
Cre (n=3)
0
5000
10000
15000 (bp)
The entire mitochondrial genome
mtDNA ND5-m.G12918A on-target
mtDNA ND5-m.G12918A
TTCCTAACAGGGTTCTACTCAAAAGACCTAATTATTGAAGCAATTAATACCTGCAA
AAGGATTGTCCCAAGATGAGTTTTC2TGGATTAATAACTTCGTTAATTATGGACGTT
mtDNA ND5-m.G12918A off-target site 1
mtDNA m.C12336T
TTCCCTAAACATAAACTCATGAGAACTTCAACAGATTATATTCTCCAACAACAA
AAGGGATTTGTATTTGAGTACTCTTGAAGTTGTCTAATATAAGAGGTTGTTGTT
mtDNA ND5-m.C12336T on-target
mtDNA ND5-m.C12336T
mtDNA ND5-m.G12341A
TTCCCTAAACATAAACTCATGAGAACTTC4AACAGATTATATTCTCCAACAACAA
AAGGGATTTGTATTTGAGTACTCTTGAAGTTGTC6TAATATAAGAGGTTGTTGTT
mtDNA ND5-m.C12336T off-target sites 1-5
mtDNA m.G12208A and m.G12211A
mtDNA m.G12220A, m.G12223A, and m.G12228A
ATATCTTTCCTACTAATTGGATGATGGTACGGACGAACAGACGCAAATACTGCAGCCCTA
TATAGAAAGGATGATTAACCTACTACCATGCCTGCTTGTCTGCGTTTATGACGTCGGGAT
mtDNA ND5-m.C12336T off-target site 6
mtDNA m.C12524T
AATAGTAGTTGCAGGAATTTTCCTACTGGTCCGATTCCACCCCCTCACGACTAATAATAAC
TTATCATCAACGTCCTTAAAAGGATGACCAGGCTAAGGTGGGGGAGTGCTGATTATTATTG

b

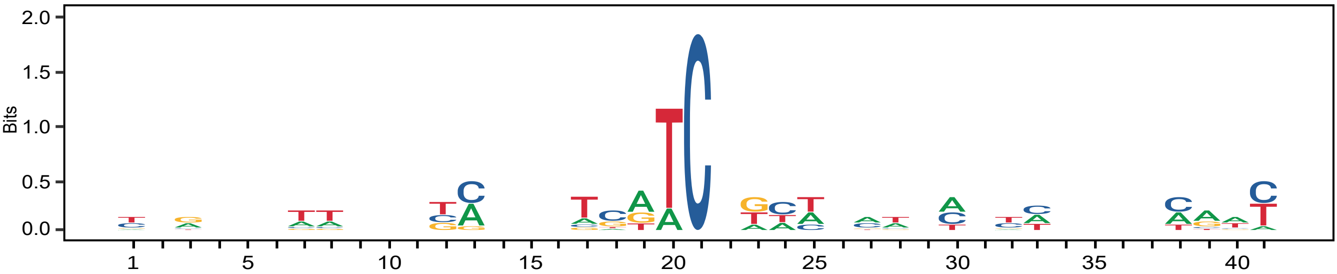

Bits
2.0
1.5
1.0
0.5
0.0
1
5
10
15
20
25
30
35
40

Supplementary Fig. S2. Off-target profile of DdCBE for mitochondrial genome editing with GOTI. a. Mitochondrial genome-wide off-target loci identified from mouse embryos injected with *ND5*-DdCBE. There is no SNVs identified in control embryos injected with only Cre mRNA but without DdCBE (bottom panel) whereas several SNVs identified in embryos injected with both Cre and *ND5*-DdCBE mRNA due to the potential off-target editing (upper five panels). **b.** Sequence logos generated from sequences with off-target C·G to T·A conversions by *ND5*-DdCBE in mitochondrial DNA genome. Bits reflect sequence conservation at a given position.

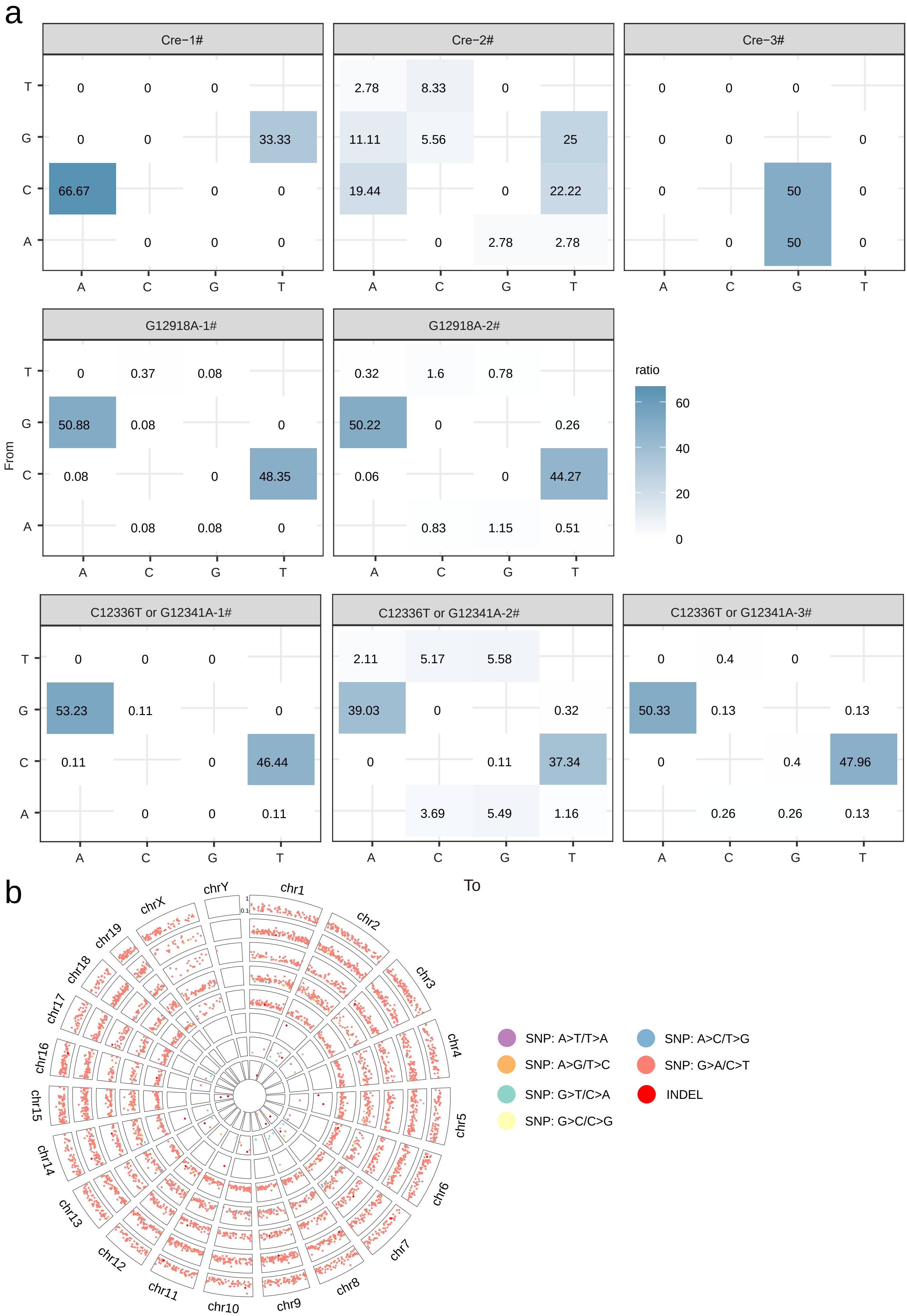
a
Cre−1#
Cre−2#
Cre−3#
G12918A-1#
G12918A-2#
C12336T or G12341A-1#
C12336T or G12341A-2#
C12336T or G12341A-3#
T
G
C
A
From
To
ratio
60
40
20
0
66.67
33.33
2.78
8.33
11.11
5.56
25
19.44
22.22
50
0.37
0.08
50.88
48.35
0.32
1.6
0.78
50.22
0.26
0.06
44.27
0.83
1.15
0.51
53.23
0.11
46.44
2.11
5.17
5.58
39.03
0.32
37.34
3.69
5.49
1.16
0.4
50.33
0.13
47.96
0.26
b
chr1
chr2
chr3
chr4
chr5
chr6
chr7
chr8
chr9
chr10
chr11
chr12
chr13
chr14
chr15
chr16
chr17
chr18
chr19
chrX
chrY
SNP: A>T/T>A
SNP: A>G/T>C
SNP: G>T/C>A
SNP: G>C/C>G
SNP: A>C/T>G
SNP: G>A/C>T
INDEL

Supplementary Fig. S3. Off-target profile of DdCBE for nuclear genome editing with GOTI. a. The distribution of mutation types in each embryo. The number in each cell indicated the proportion of a certain mutation type among all identified SNVs, and deeper colors represented higher proportions. **b.** Distribution of SNVs or INDELs in the mouse genome in Cre and *ND5*-DdCBE-treated samples. Genome-wide distribution SNVs or INDELs from outer circle to inner circle were identified from m.G12918A-1#, m.G12918A-2#, m.C12336T-1#, m.C12336T-2#, m.C12336T-3#, Cre-1#, Cre-2#, and Cre-3# embryos.

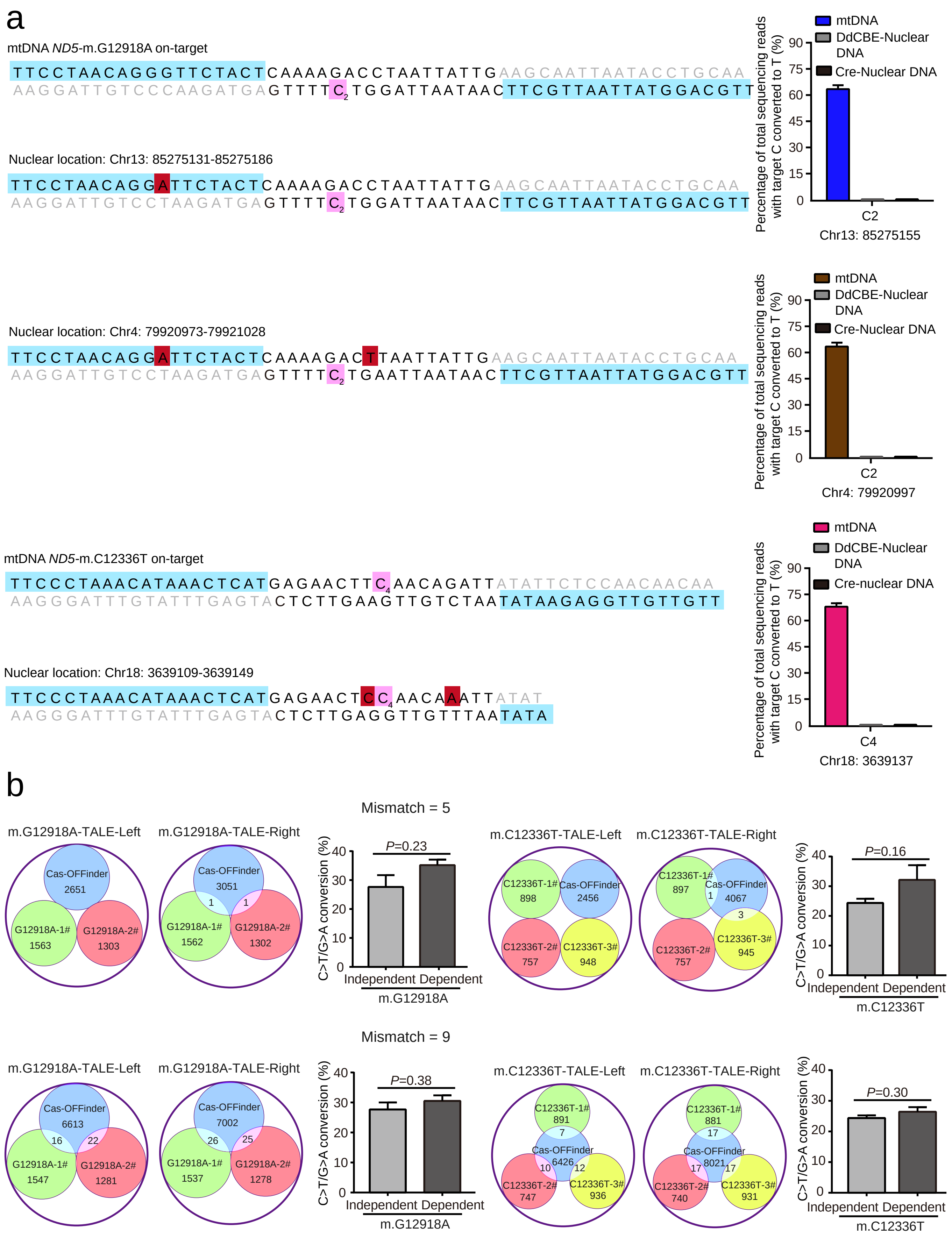
a
mtDNA ND5-m.G12918A on-target
TTCCTAACAGGGTTCTACTCAAAAGACCTAATTATTGAAGCAATTAATACCTGCAA
AAGGATTGTCCCAAGATGAGTTTTC2TGGATTAATAACTTCGTTAATTATGGACGTT
Nuclear location: Chr13: 85275131-85275186
TTCCTAACAGGATTCTACTCAAAAGACCTAATTATTGAAGCAATTAATACCTGCAA
AAGGATTGTCCTAAGATGAGTTTTC2TGGATTAATAACTTCGTTAATTATGGACGTT
Percentage of total sequencing reads with target C converted to T (%)
mtDNA
DdCBE-Nuclear DNA
Cre-Nuclear DNA
C2
Chr13: 85275155
Nuclear location: Chr4: 79920973-79921028
TTCCTAACAGGATTCTACTCAAAAGACTTAATTATTGAAGCAATTAATACCTGCAA
AAGGATTGTCCTAAGATGAGTTTTC2TGAATTAATAACTTCGTTAATTATGGACGTT
C2
Chr4: 79920997
mtDNA ND5-m.C12336T on-target
TTCCCTAAACATAAACTCATGAGAACTTC4AACAGATTATATTCTCCAACAACAA
AAGGGATTTGTATTTGAGTACTCTTGAAGTTGTCTAATTATAAGAGGTTGTTGTT
Nuclear location: Chr18: 3639109-3639149
TTCCCTAAACATAAACTCATGAGAACTCC4AACAAATTATAT
AAGGGATTTGTATTTGAGTACTCTTGAGGTTGTTTAATTATA
Cre-nuclear DNA
C4
Chr18: 3639137
b
Mismatch = 5
m.G12918A-TALE-Left
Cas-OFFinder 2651
G12918A-1# 1563
G12918A-2# 1303
m.G12918A-TALE-Right
Cas-OFFinder 3051
G12918A-1# 1562
G12918A-2# 1302
C>T/G>A conversion (%)
P=0.23
Independent Dependent
m.G12918A
m.C12336T-TALE-Left
C12336T-1# 898
Cas-OFFinder 2456
C12336T-2# 757
C12336T-3# 948
m.C12336T-TALE-Right
C12336T-1# 897
Cas-OFFinder 4067
C12336T-2# 757
C12336T-3# 945
P=0.16
m.C12336T
Mismatch = 9
m.G12918A-TALE-Left
Cas-OFFinder 6613
G12918A-1# 1547
G12918A-2# 1281
m.G12918A-TALE-Right
Cas-OFFinder 7002
G12918A-1# 1537
G12918A-2# 1278
P=0.38
m.C12336T-TALE-Left
C12336T-1# 891
Cas-OFFinder 6426
C12336T-2# 747
C12336T-3# 936
m.C12336T-TALE-Right
C12336T-1# 881
Cas-OFFinder 8021
C12336T-2# 740
C12336T-3# 931
P=0.30

Supplementary Fig. S4. Off-target analysis of DdCBEs on nuclear DNA in mouse E14.5 fetuses. a. Left: the on-target editing sites on mtDNA and the corresponding nuclear DNA sequence with the greatest homology are shown for *ND5*-m.G12918A-DdCBE and *ND5*-m.C12336T-DdCBE. TALE binding sites begin at N0 and are shown in blue. Target cytosines are in purple. Nucleotide mismatches between the mtDNA and nuclear mitochondrial DNA segments (NUMTs) are in red. Right: editing efficiencies are measured by next-generation sequencing. **b.** Overlap and efficiency bias analysis among SNVs detected by GOTI with predicted off-targets by Cas-OFFinder. Data are presented as means ± SEM. *P* values were evaluated with the unpaired student's *t*-test (two-tailed).

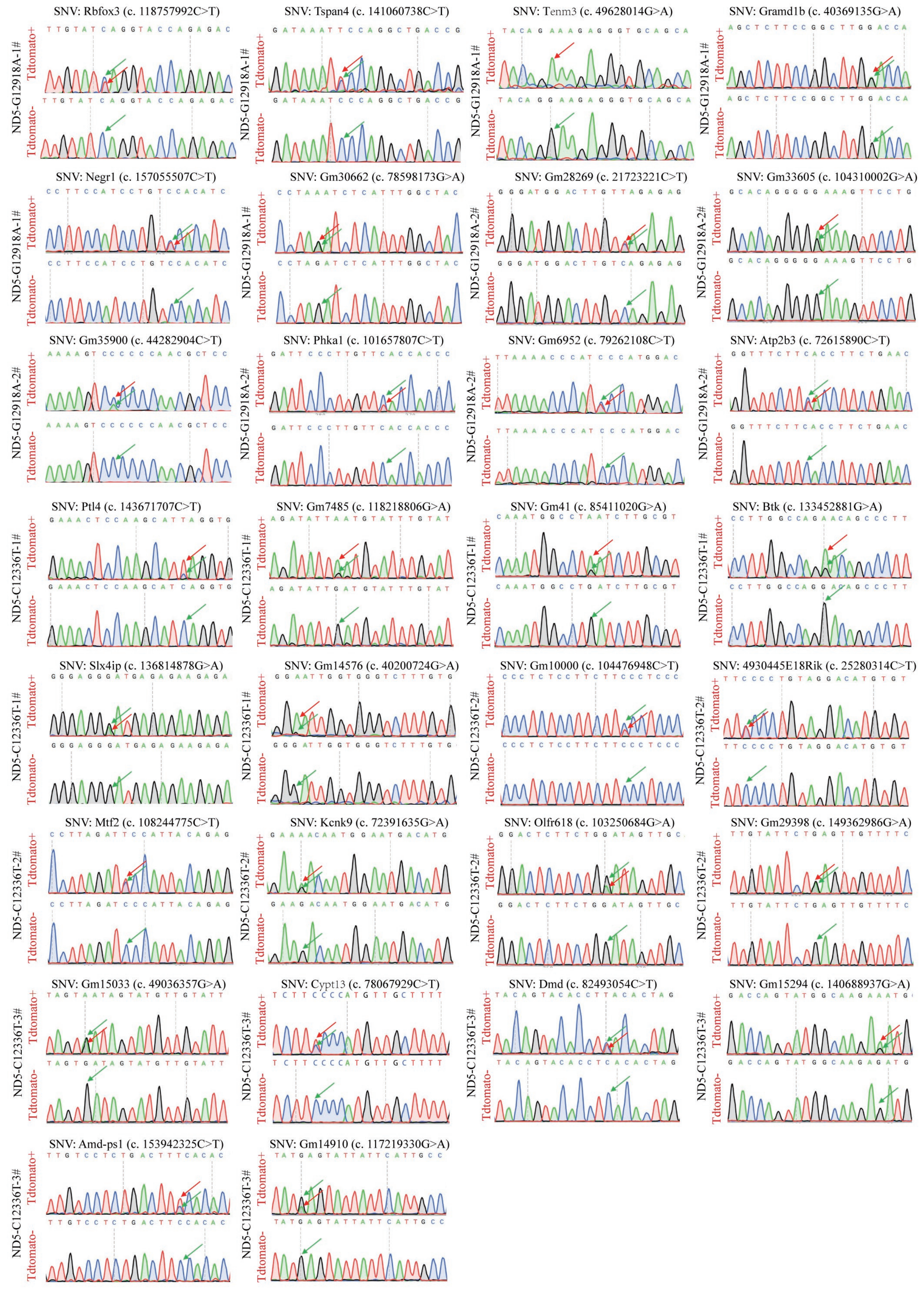
SNV: Rbfox3 (c. 118757992C>T)
SNV: Tspan4 (c. 141060738C>T)
SNV: Tenm3 (c. 49628014G>A)
SNV: Gramd1b (c. 40369135G>A)
SNV: Negr1 (c. 157055507C>T)
SNV: Gm30662 (c. 78598173G>A)
SNV: Gm28269 (c. 21723221C>T)
SNV: Gm33605 (c. 104310002G>A)
SNV: Gm35900 (c. 44282904C>T)
SNV: Phka1 (c. 101657807C>T)
SNV: Gm6952 (c. 79262108C>T)
SNV: Atp2b3 (c. 72615890C>T)
SNV: Ptl4 (c. 143671707C>T)
SNV: Gm7485 (c. 118218806G>A)
SNV: Gm41 (c. 85411020G>A)
SNV: Btk (c. 133452881G>A)
SNV: Slx4ip (c. 136814878G>A)
SNV: Gm14576 (c. 40200724G>A)
SNV: Gm10000 (c. 104476948C>T)
SNV: 4930445E18Rik (c. 25280314C>T)
SNV: Mtf2 (c. 108244775C>T)
SNV: Kcnk9 (c. 72391635G>A)
SNV: Olfr618 (c. 103250684G>A)
SNV: Gm29398 (c. 149362986G>A)
SNV: Gm15033 (c. 49036357G>A)
SNV: Cypt13 (c. 78067929C>T)
SNV: Dmd (c. 82493054C>T)
SNV: Gm15294 (c. 140688937G>A)
SNV: Amd-ps1 (c. 153942325C>T)
SNV: Gm14910 (c. 117219330G>A)
ND5-G12918A-1#
ND5-G12918A-2#
ND5-C12336T-1#
ND5-C12336T-2#
ND5-C12336T-3#
Tdtomato+
Tdtomato-

Supplementary Fig. S5. Sanger sequencing chromatograms of SNVs detected by WGS in DdCBE-treated embryos. SNVs from the indicated samples were validated by Sanger sequencing. Green arrows indicate wild-type nucleotide, and red arrows indicate mutated nucleotide. Primers were listed in Supplementary Table S2.

Supplementary Table S1. Summary of the numbers of mouse Ai9×C57BL/6 embryos used and mutants obtained by injection of the DdCBEs.

Type of Mutagenesis	Number of injected Ai9×C57 embryos	Number of transferred Ai9×C57 embryos	Total E14.5 pups (%)	Number of edited E14.5 pups (%)	Mutation load (%)
ND5-m.G12918A	53	45	11 (24.4)	9 (81.8)	7.1-35.3
ND5-m.C12336T	50	45	14 (31.1)	11 (78.6)	8.4-46.1
Cre	35	30	9 (30.0)	0 (0)	NA

Supplementary Table S2. The primers used in this study.

List of PCR primers used for Sanger sequencing of off-target sites		
Off-target sites	PCR primer	Sequence (5'-3')
Rbfox3	Mus mito-*ND5* G12918A-1#-1F	ATGCTGGGTAATGGATGT
	Mus mito-*ND5* G12918A-1#-1R	TTGGCTCTTTCTTAGGTGT
Tspan4	Mus mito-*ND5* G12918A-1#-2F	CACGGGTCACAAGGGTTT
	Mus mito-*ND5* G12918A-1#-2R	GGACGGAAGGCAGAACAA
Tenm3	Mus mito-*ND5* G12918A-1#-3F	TTAGCGATATGGAAAGGT
	Mus mito-*ND5* G12918A-1#-3R	TGAAATAATGACGGGACT
Gramd1b	Mus mito-*ND5* G12918A-1#-4F	GTTCACCCTAAGCGATGT
	Mus mito-*ND5* G12918A-1#-4R	CTGGTAATGCCCTGTTTT
Negr1	Mus mito-*ND5* G12918A-1#-5F	AAATGGGAATGATTAGGG
	Mus mito-*ND5* G12918A-1#-5R	TGAAGGGAAGCAGTAGAG
Gm30662	Mus mito-*ND5* G12918A-1#-6F	TACCAATGCCTTCCAGAC
	Mus mito-*ND5* G12918A-1#-6R	GCACAGGGATGAAGATGA
Gm28269	Mus mito-*ND5* G12918A-2#-1F	GTCCAGCTAGTCCTACAA
	Mus mito-*ND5* G12918A-2#-1R	ACAACAAGTACAAACCCT
Gm33605	Mus mito-*ND5* G12918A-2#-2F	GCAAATTGTATCTTGGCTAT
	Mus mito-*ND5* G12918A-2#-2R	TTTCTGGAGTCCACCTTC
Gm35900	Mus mito-*ND5* G12918A-2#-3F	TGTGGTCTGGAGGGTCAA
	Mus mito-*ND5* G12918A-2#-3R	ATCAGTGGAAACAGAGGC
Phka1	Mus mito-*ND5* G12918A-2#-4F	ACAGAAGTGACTTGGCTCAA
	Mus mito-*ND5* G12918A-2#-4R	GGTCCCATACCCAGTGTT
Gm6952	Mus mito-*ND5* G12918A-2#-5F	GAAGAAAGGAGGTAGAGG
	Mus mito-*ND5* G12918A-2#-5R	ATATTTGGCTGTGGGTAT
Atp2b3	Mus mito-*ND5* G12918A-2#-6F	TCTGGGAATCCTGAGATA
	Mus mito-*ND5* G12918A-2#-6R	CAGTGGGAACATAGCAAG
Ptl4	Mus mito-*ND5* C12336T-1#-1F	AGGAGGGAAGTTGTTAGA
	Mus mito-*ND5* C12336T-1#-1R	TTCATTGGCATTACAGAG
Gm7485	Mus mito-*ND5* C12336T-1#-2F	ACCCTTTCCCAATCTGTA
	Mus mito-*ND5* C12336T-1#-2R	TGACCTCAAGTTGCCCTA
Gm41	Mus mito-*ND5* C12336T-1#-3F	AGAGCCAACACTCAAACA
	Mus mito-*ND5* C12336T-1#-3R	GGCAGTAGTCCCAAGATG
Btk	Mus mito-*ND5* C12336T-1#-4F	CATCTCGTCCTGTTTGAC
	Mus mito-*ND5* C12336T-1#-4R	CTATGGTTATGTGGGTGC
Slx4ip	Mus mito-*ND5* C12336T-1#-5F	ACCTAGCCTGAGCAAATC
	Mus mito-*ND5* C12336T-1#-5R	TCAGCCAGCTAAGGAGTT
Gm14576	Mus mito-*ND5* C12336T-1#-6F	GGGTGATAGAGTTTCATAG
	Mus mito-*ND5* C12336T-1#-6R	TACAGAAGAGGGAGTAGAG
Gm10000	Mus mito-*ND5* C12336T-2#-1F	GTGAACAGTATCCCAGTCG
	Mus mito-*ND5* C12336T-2#-1R	ACAGCCAAGGGTAGTAGAG

4930445E18Rik	Mus mito-*ND5* C12336T-2#-2F	CCAAGGTCCAGCTATCAA
	Mus mito-*ND5* C12336T-2#-2R	GGAAGGAGGGCTTATTTT
Mtf2	Mus mito-*ND5* C12336T-2#-3F	TGTCTGAGCGTGTACTTT
	Mus mito-*ND5* C12336T-2#-3R	CCTAATCTTAGATACCCATAAC
Kcnk9	Mus mito-*ND5* C12336T-2#-4F	AGAAGGATGAGTGCCTAG
	Mus mito-*ND5* C12336T-2#-4R	AGAAGGAGAAAGGGTATG
Olfr618	Mus mito-*ND5* C12336T-2#-5F	TTCCAGCAAAGAAAGACC
	Mus mito-*ND5* C12336T-2#-5R	ATCCCATTCATTAAGTCC
Gm29398	Mus mito-*ND5* C12336T-2#-6F	CTCTGCTTCTTGCCTTAC
	Mus mito-*ND5* C12336T-2#-6R	CTCATAATCCAGCCCATC
Gm15033	Mus mito-*ND5* C12336T-3#-1F	GTCTGCGGTGTTAGGTGT
	Mus mito-*ND5* C12336T-3#-1R	AGTGCTGCTCAATGAAAT
Cypt13	Mus mito-*ND5* C12336T-3#-2F	GCATTAGTTCTTGCCTCCAA
	Mus mito-*ND5* C12336T-3#-2R	TTCTGCCACCCTCCTCAC
Dmd	Mus mito-*ND5* C12336T-3#-3F	TTCCTCACCTCCACCTTA
	Mus mito-*ND5* C12336T-3#-3R	GGACGTTCCCTACAGATA
Gm15294	Mus mito-*ND5* C12336T-3#-4F	GCTCCCATTGTTGAAGAT
	Mus mito-*ND5* C12336T-3#-4R	TGCTGTAAAGAGGGTGTT
Amd-ps1	Mus mito-*ND5* C12336T-3#-5F	CTTGCTGCTCTTGAAATA
	Mus mito-*ND5* C12336T-3#-5R	ATCAGACAGGAGGGTTAG
Gm14910	Mus mito-*ND5* C12336T-3#-6F	TTGCCATAGTCCACATCC
	Mus mito-*ND5* C12336T-3#-6R	TCCAGTCCCATACACCAG
**List of PCR primers used for Sanger sequencing of on-target sites**		
On-target sites	PCR primer	Sequence (5’-3’)
G12918A	Mus mito-*ND5* G12918A-F	GCCAACTAGGCCTGATAATAG
	Mus mito-*ND5* G12918A-R	GGCGTTTGATTGGGTTTATG
C12336T	Mus mito-*ND5* C12366T-F	CACCTCAGCCAACAACAT
	Mus mito-*ND5* C12366T-R	TTGGGTGAGAGCACAAATAG
**List of PCR primers used for Targeted deep sequencing of on-target sites**		
On-target sites	PCR primer	Sequence (5’-3’)
G12918A	Mus mito-*ND5* G12918A-F	GTACNNNNNNNNTCATACCATTCACATCATCA
	Mus mito-*ND5* G12918A-R	CGCGGTTTTGTTATTGTTAC
C12336T	Mus mito-*ND5* C12366T-F	GTACNNNNNNNNCAGCCCTACAAGCAATCCTC
	Mus mito-*ND5* C12366T-R	TGGAGGCCAAATTGTGCTGA

Supplementary sequence:

DdCBE domains are annotated as: yellow for MTS, italics for linker, cyan for HA or FLAG tag, purple for N&C-terminal domain, underlined for RVD, red for half of DddAtox, green for UGI.

ND5-m.G12918A-DdCBE: Left-SOD2 MTS-3×HA-mitoTALE-G1397-DddA$_{tox}$-C-1×UGI

MALSRAVCGTSRQLAPVLGYLGSRQKHSLPDYPYDVPDYAGYPYDVPDYAG
YPYDVPDYAGIRIQDLRTLGYSQQQQEKIKPKVRSTVAQHHEALVGHGFTHA
HIVALSQHPAALGTVAVKYQDMIAALPEATHEAIVGVGKQWSGARALEALL
TVAGELRGPPLQLDTGQLLKIAKRGGVTAVEAVHAWRNALTGAPLNLTPAQ
VVAIASNGGGKQALETVQRLLPVLCQAHGLTPDQVVAIASNNGGKQALETV
QRLLPVLCQAHGLTPAQVVAIASHDGGKQALETVQRLLPVLCQAHGLTPAQ
VVAIASNIGGKQALETVQRLLPVLCQAHGLTPAQVVAIASNNGGKQALETVQ
RLLPVLCQDHGLTPAQVVAIASNNGGKQALETVQRLLPVLCQAHGLTPDQV
VAIASNGGGKQALETVQRLLPVLCQAHGLTPDQVVAIASNIGGKQALETVQR
LLPVLCQDHGLTPDQVVAIASNGGGKQALETVQRLLPVLCQAHGLTPEQVV
AIASNGGGKQALETVQRLLPVLCQAHGLTPAQVVAIASNIGGKQALETVQRL
LPVLCQDHGLTPAQVVAIASNIGGKQALETVQRLLPVLCQAHGLTPEQVVAI
ASNGGGKQALETVQRLLPVLCQAHGLTPAQVVAIASNGGGKQALETVQRLL
PVLCQAHGLTPDQVVAIASNNGGKQALETVQRLLPVLCQDHGLTPDQVVAIA
SHDGGKQALETVQRLLPVLCQAHGLTPAQVVAIASNGGGKQALETVQRLLP
VLCQDHGLTPEQVVAIASNGGGKQALESIVAQLSRPDPALAALTNDHLVALA
CLGGRPALDAVKKGLGGS*GS*AIPVKRGATGETKVFTGNSNSPKSPTKGGC*SG*
*GS*TNLSDIIEKETGKQLVIQESILMLPEEVEEVIGNKPESDILVHTAYDESTDEN
VMLLTSDAPEYKPWALVIQDSNGENKIKML*

ND5- m.G12918A-DdCBE: Right-COX8A MTS-3×FLAG-mitoTALE-G1397-DddA$_{tox}$-N-1×UGI

MASVLTPLLLRGLTGSARRLPVPRAKIHSLDYKDHDGDYKDHDIDYKDDDD
KGIRIQDLRTLGYSQQQQEKIKPKVRSTVAQHHEALVGHGFTHAHIVALSQHP
AALGTVAVKYQDMIAALPEATHEAIVGVGKQWSGARALEALLTVAGELRGP
PLQLDTGQLLKIAKRGGVTAVEAVHAWRNALTGAPLNLTPAQVVAIASNGG
GKQALETVQRLLPVLCQDHGLTPAQVVAIASHDGGKQALETVQRLLPVLCQ
AHGLTPEQVVAIASHDGGKQALETVQRLLPVLCQAHGLTPEQVVAIASNGGG
KQALETVQRLLPVLCQAHGLTPAQVVAIASNIGGKQALETVQRLLPVLCQDH
GLTPAQVVAIASNIGGKQALETVQRLLPVLCQAHGLTPEQVVAIASHDGGKQ
ALETVQRLLPVLCQAHGLTPAQVVAIASNIGGKQALETVQRLLPVLCQAHGL
TPAQVVAIASNNGGKQALETVQRLLPVLCQAHGLTPAQVVAIASNNGGKQA
LETVQRLLPVLCQDHGLTPAQVVAIASNNGGKQALETVQRLLPVLCQDHGLT
PDQVVAIASNGGGKQALETVQRLLPVLCQAHGLTPEQVVAIASNGGGKQAL
ETVQRLLPVLCQAHGLTPDQVVAIASHDGGKQALETVQRLLPVLCQDHGLTP
DQVVAIASNGGGKQALETVQRLLPVLCQAHGLTPEQVVAIASNIGGKQALET

VQRLLPVLCQAHGLTPAQVVAIASHDGGKQALETVQRLLPVLCQDHGLTPEQ
VVAIASNGGGKQALESIVAQLSRPDPALAALTNDHLVALACLGGRPALDAVK
KGLGGS*GS*GSYALGPYQISAPQLPAYNGQTVGTFYYVNDAGGLESKVFSSGG
PTPYPNYANAGHVEGQSALFMRDNGISEGLVFHNNPEGTCGFCVNMTETLLP
ENAKMTVVPPEG*SGGS*TNLSDIIEKETGKQLVIQESILMLPEEVEEVIGNKPES
DILVHTAYDESTDENVMLLTSDAPEYKPWALVIQDSNGENKIKML*

ND5- m.C12336T or m.G12341A-DdCBE: Left-SOD2 MTS-3×HA-mitoTALE-G1333-DddA$_{tox}$-N-1×UGI

MALSRAVCGTSRQLAPVLGYLGSRQKHSLPDYPYDVPDYAGYPYDVPDYAG
YPYDVPDYAGIRIQDLRTLGYSQQQQEKIKPKVRSTVAQHHEALVGHGFTHA
HIVALSQHPAALGTVAVKYQDMIAALPEATHEAIVGVGKQWSGARALEALL
TVAGELRGPPLQLDTGQLLKIAKRGGVTAVEAVHAWRNALTGAPLNLTPAQ
VVAIASNGGGKQALETVQRLLPVLCQDHGLTPAQVVAIASHDGGKQALETV
QRLLPVLCQAHGLTPEQVVAIASHDGGKQALETVQRLLPVLCQAHGLTPDQV
VAIASHDGGKQALETVQRLLPVLCQAHGLTPDQVVAIASNGGGKQALETVQ
RLLPVLCQDHGLTPAQVVAIASNIGGKQALETVQRLLPVLCQDHGLTPDQVV
AIASNIGGKQALETVQRLLPVLCQAHGLTPDQVVAIASNIGGKQALETVQRLL
PVLCQAHGLTPAQVVAIASHDGGKQALETVQRLLPVLCQAHGLTPAQVVAIA
SNIGGKQALETVQRLLPVLCQDHGLTPAQVVAIASNGGGKQALETVQRLLPV
LCQDHGLTPDQVVAIASNIGGKQALETVQRLLPVLCQDHGLTPDQVVAIASNI
GGKQALETVQRLLPVLCQAHGLTPDQVVAIASNIGGKQALETVQRLLPVLCQ
AHGLTPAQVVAIASHDGGKQALETVQRLLPVLCQAHGLTPAQVVAIASNGG
GKQALETVQRLLPVLCQDHGLTPEQVVAIASHDGGKQALETVQRLLPVLCQ
AHGLTPAQVVAIASNIGGKQALETVQRLLPVLCQDHGLTPEQVVAIASNGGG
KQALESIVAQLSRPDPALAALTNDHLVALACLGGRPALDAVKKGLGGS*GS*GS
YALGPYQISAPQLPAYNGQTVGTFYYVNDAGGLESKVFSSGG*SGGS*TNLSDII
EKETGKQLVIQESILMLPEEVEEVIGNKPESDILVHTAYDESTDENVMLLTSDA
PEYKPWALVIQDSNGENKIKML*

ND5- m.C12336T or m.G12341A-DdCBE: Right-COX8A MTS-3×FLAG-mitoTALE-G1333-DddA$_{tox}$-C-1×UGI

MASVLTPLLLRGLTGSARRLPVPRAKIHSLDYKDHDGDYKDHDIDYKDDDD
KGIRIQDLRTLGYSQQQQEKIKPKVRSTVAQHHEALVGHGFTHAHIVALSQHP
AALGTVAVKYQDMIAALPEATHEAIVGVGKQWSGARALEALLTVAGELRGP
PLQLDTGQLLKIAKRGGVTAVEAVHAWRNALTGAPLNLTPAQVVAIASNGG
GKQALETVQRLLPVLCQAHGLTPDQVVAIASNNGGKQALETVQRLLPVLCQ
AHGLTPAQVVAIASNGGGKQALETVQRLLPVLCQDHGLTPAQVVAIASNGG
GKQALETVQRLLPVLCQAHGLTPDQVVAIASNNGGKQALETVQRLLPVLCQ
AHGLTPAQVVAIASNGGGKQALETVQRLLPVLCQDHGLTPEQVVAIASNGG
GKQALETVQRLLPVLCQAHGLTPAQVVAIASNNGGKQALETVQRLLPVLCQ
DHGLTPDQVVAIASNNGGKQALETVQRLLPVLCQAHGLTPDQVVAIASNIGG

KQALETVQRLLPVLCQDHGLTPAQVVAIASNNGGKQALETVQRLLPVLCQD
HGLTPAQVVAIASNIGGKQALETVQRLLPVLCQAHGLTPEQVVAIASNIGGKQ
ALETVQRLLPVLCQAHGLTPDQVVAIASNGGGKQALETVQRLLPVLCQDHG
LTPDQVVAIASNIGGKQALETVQRLLPVLCQAHGLTPEQVVAIASNGGGKQA
LESIVAQLSRPDPALAALTNDHLVALACLGGRPALDAVKKGLGGS*GS*PTPYP
NYANAGHVEGQSALFMRDNGISEGLVFHNNPEGTCGFCVNMTETLLPENAK
MTVVPPEGAIPVKRGATGETKVFTGNSNSPKSPTKGGC*SGGS*TNLSDIIEKETG
KQLVIQESILMLPEEVEEVIGNKPESDILVHTAYDESTDENVMLLTSDAPEYKP
WALVIQDSNGENKIKML*
